# Bis(2-fluoro­benzoato-κ^2^ *O*,*O*′)bis­(1,10-phenanthroline-κ^2^ *N*,*N*′)lead(II) dihydrate

**DOI:** 10.1107/S1600536809035016

**Published:** 2009-09-05

**Authors:** Bi-Song Zhang

**Affiliations:** a College of Materials Science and Chemical Engineering, Jinhua College of Profession and Technology, Jinhua, Zhejiang 321017, People’s Republic of China

## Abstract

In the title compound, [Pb(C_7_H_4_FO_2_)_2_(C_12_H_8_N_2_)_2_]·2H_2_O, the Pb^II^ atom is coordinated by four N atoms from two bidentate chelating 1,10-phenanthroline (phen) ligands and four O atoms from two 2-fluoro­benzoate ligands in an irregular polyhedral coordination geometry. Two carboxyl­ate O atoms and one F atom are each disordered over two sites with occupancy factors of 0.60 and 0.40. The dihedral angle between the two phen ligands is 89.9 (1)°. The mean inter­planar distances are alternatively of 3.44 (3) and 3.45 (3) Å, indicating π–π stacking inter­actions between the neighboring phen ligands. In the crystal, O—H⋯O, O—H⋯F and C—H⋯O hydrogen bonds link the complex mol­ecules and uncoordinated water mol­ecules into a supra­molecular network.

## Related literature

For other complexes with a 2(or 4)-fluoro­benzoate ligand, see: Ye & Zhang (2009 ▶); Zhang *et al.* (2005 ▶). For related structures, see: Zhang (2004 ▶, 2005 ▶, 2006*a* ▶,*b* ▶,*c* ▶).
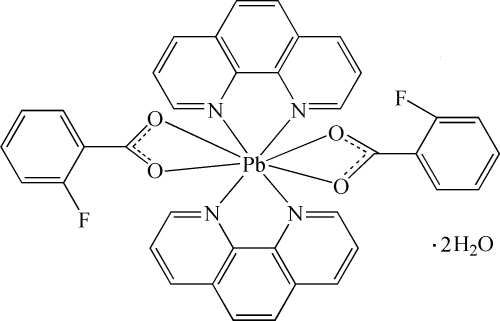


## Experimental

### 

#### Crystal data

[Pb(C_7_H_4_FO_2_)_2_(C_12_H_8_N_2_)_2_]·2H_2_O
*M* *_r_* = 881.83
Triclinic, 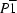

*a* = 11.406 (2) Å
*b* = 12.510 (3) Å
*c* = 13.771 (3) Å
α = 95.11 (3)°
β = 114.39 (3)°
γ = 101.72 (3)°
*V* = 1719.0 (9) Å^3^
*Z* = 2
Mo *K*α radiation
μ = 4.97 mm^−1^
*T* = 290 K
0.29 × 0.18 × 0.17 mm

#### Data collection

Rigaku R-AXIS RAPID diffractometer
Absorption correction: multi-scan (*ABSCOR*; Higashi, 1995 ▶) *T*_min_ = 0.353, *T*_max_ = 0.428
13556 measured reflections
6018 independent reflections
4795 reflections with *I* > 2σ(*I*)
*R*_int_ = 0.060

#### Refinement

*R*[*F* ^2^ > 2σ(*F* ^2^)] = 0.042
*wR*(*F* ^2^) = 0.136
*S* = 1.22
6018 reflections
484 parameters
H-atom parameters constrained
Δρ_max_ = 2.12 e Å^−3^
Δρ_min_ = −2.69 e Å^−3^

### 

Data collection: *PROCESS-AUTO* (Rigaku, 1998 ▶); cell refinement: *PROCESS-AUTO*; data reduction: *PROCESS-AUTO*; program(s) used to solve structure: *SHELXS97* (Sheldrick, 2008 ▶); program(s) used to refine structure: *SHELXL97* (Sheldrick, 2008 ▶); molecular graphics: *SHELXTL* (Sheldrick, 2008 ▶); software used to prepare material for publication: *SHELXTL*.

## Supplementary Material

Crystal structure: contains datablocks I, global. DOI: 10.1107/S1600536809035016/hy2223sup1.cif

Structure factors: contains datablocks I. DOI: 10.1107/S1600536809035016/hy2223Isup2.hkl

Additional supplementary materials:  crystallographic information; 3D view; checkCIF report

## Figures and Tables

**Table 1:** Selected bond lengths (Å)

Pb1—N1	2.675 (9)
Pb1—N2	2.644 (8)
Pb1—N3	2.622 (9)
Pb1—N4	2.566 (8)
Pb1—O1	2.788 (16)
Pb1—O1′	2.95 (3)
Pb1—O2	2.880 (18)
Pb1—O2′	2.77 (3)
Pb1—O3	2.670 (8)
Pb1—O4	2.777 (9)

**Table 2:** Hydrogen-bond geometry (Å, °)

*D*—H⋯*A*	*D*—H	H⋯*A*	*D*⋯*A*	*D*—H⋯*A*
O5—H5*A*⋯O1′^i^	0.85	2.34	3.027 (4)	138
O5—H5*A*⋯F1^ii^	0.85	2.33	2.801 (5)	116
O5—H5*A*⋯O2′^i^	0.85	2.51	3.313 (6)	158
O5—H5*B*⋯O2′^ii^	0.85	2.05	2.789 (3)	146
O5—H5*B*⋯O1^ii^	0.85	1.99	2.792 (6)	158
O6—H6*A*⋯O4	0.85	2.08	2.807 (11)	143
O6—H6*B*⋯O2^iii^	0.85	2.03	2.795 (5)	149
O6—H6*B*⋯O1′^iii^	0.85	2.17	2.889 (5)	143
O7—H7*A*⋯O5	0.85	1.97	2.75 (2)	152
O7—H7*B*⋯O6^iv^	0.85	2.29	2.810 (2)	120
C8—H8⋯O5	0.93	2.54	3.344 (34)	145
C16—H16⋯O3^v^	0.93	2.54	3.422 (19)	158
C21—H21⋯O1	0.93	2.44	3.106 (82)	127

Symmetry codes: (i) 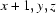; (ii) 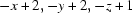; (iii) 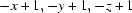; (iv) 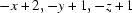; (v) 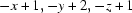.

**Fig. 1.**
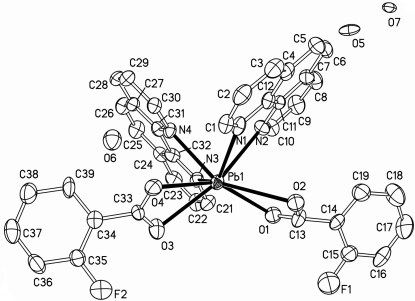
Molecular structure of the title compound. Displacement ellipsoids are drawn at the 50% probability level. H atoms and minor disordered O1', O2', F1' atoms have been omitted for clarity.

**Fig. 2.**
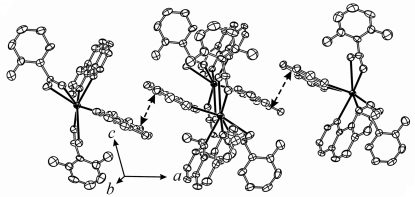
The π–π stacking interactions (dashed double arrows), with the mean interplanar distances of 3.44 (3) and 3.45 (3) Å.

## supplementary crystallographic information

### Comment

The synthesis was originally directed to repeat the synthesis of [Pb(C_7_H_4_FO_2_)_2_(C_12_H_8_N_2_)_2_(H_2_O)_0.5_].2H_2_O, (I), (Ye & Zhang, 2009). The title compound was unintentionally obtained and structurally related to (I).

The title compound (Fig .1) shows a structure similar to (I) and to those of the complexes with halobenzoate ligands, X–C_6_H_4_COO^-^, where X is F, Cl, Br and I (Zhang, 2004, 2005, 2006a,b,c; Zhang *et al.*, 2005). The asymmetric unit of the title compound consists of a [Pb(C_7_H_4_FO_2_)_2_(C_12_H_8_N_2_)_2_] complex molecule and two uncoordinated water molecules. The Pb^II^ atom is coordinated by four N atoms from two bidentate chelating phen ligands and four O atoms from two 2-fluorobenzoate ligands in an irregular polyhedral coordination geometry, with Pb—N bond lengths in the range of 2.566 (8) to 2.675 (9)Å and Pb—O bond lengths in the range of 2.670 (8) to 2.95 (3)Å (Table 1). The dihedral angle of the two phen ligands is 89.9 (1)°, as distinct from (I) (0.0 (2)°). The mean interplanar distances are alternatively of 3.44 (3) and 3.45 (3) Å, indicating π–π stacking interactions between the neighboring phen ligands (Fig. 2). O—H···O, O—H···F and C—H···O hydrogen bonds are present (Table 2 ). A combination of the π–π stacking interactions and hydrogen bonds leads to a supramolecular network.

### Experimental

Pb(NO_3_)_2_ (0.331 g, 1.00 mmol) was dissolved in appropriate amount of water, and then 1M Na_2_CO_3_ solution was added. PbCO_3_ was obtained by filtration, which was then washed with distilled water for 5 times. The freshly prepared PbCO_3_, phen (0.050 g, 0.25 mmol), 2-fluorobenzoic acid (0.036 g, 0.25 mmol), CH_3_OH/H_2_O (v/v = 1:2, 15 ml) were mixed and stirred for 2 h. Subsequently, the resulting cream suspension was heated in a 23 ml Teflon-lined stainless steel autoclave at 433 K for 5 d. After the autoclave was cooled to room temperature, the solid was filtered off. The resulting filtrate was allowed to stand at room temperature, and evaporation for 2 weeks afforded colorless transparent block single crystals.

### Refinement

H atoms on C atoms were positioned geometrically and refined as riding atoms, with C—H = 0.93 Å and with *U*_iso_(H) = 1.2*U*_eq_(C). H atoms of water molecules were located in a difference Fourier map and refined with restraints of O—H = 0.85 (1) Å and *U*_iso_(H) = 1.5*U*_eq_(O). Two carboxylate O atoms (O1 and O2) and one F atom (F1) are each disordered over two sites with occupancy factors of 0.60 and 0.40. Two water molecules (O5 and O7) are half-occupied. The largest peak in the final difference Fourier map is 1.36 Å from atom Pb1 and the deepest hole is 0.97 Å from atom Pb1.

### Crystal data

**Table:**

[Pb(C_7_H_4_FO_2_)_2_(C_12_H_8_N_2_)_2_]·2H_2_O	*Z* = 2
*M**_r_* = 881.83	*F*(000) = 864
Triclinic, *P*1	*D*_x_ = 1.704 Mg m^−^^3^
Hall symbol: -P 1	Mo *K*α radiation, λ = 0.71073 Å
*a* = 11.406 (2) Å	Cell parameters from 12091 reflections
*b* = 12.510 (3) Å	θ = 3.0–25.0°
*c* = 13.771 (3) Å	µ = 4.97 mm^−^^1^
α = 95.11 (3)°	*T* = 290 K
β = 114.39 (3)°	Block, colorless
γ = 101.72 (3)°	0.29 × 0.18 × 0.17 mm
*V* = 1719.0 (9) Å^3^	

### Data collection

**Table:**

Rigaku R-AXIS RAPID diffractometer	6018 independent reflections
Radiation source: rotating anode	4795 reflections with *I* > 2σ(*I*)
graphite	*R*_int_ = 0.060
ω scans	θ_max_ = 25.0°, θ_min_ = 3.0°
Absorption correction: multi-scan (*ABSCOR*; Higashi, 1995)	*h* = −13→13
*T*_min_ = 0.353, *T*_max_ = 0.428	*k* = −14→14
13556 measured reflections	*l* = −16→16

### Refinement

**Table:**

Refinement on *F*^2^	Primary atom site location: structure-invariant direct methods
Least-squares matrix: full	Secondary atom site location: difference Fourier map
*R*[*F*^2^ > 2σ(*F*^2^)] = 0.042	Hydrogen site location: inferred from neighbouring sites
*wR*(*F*^2^) = 0.136	H-atom parameters constrained
*S* = 1.22	*w* = 1/[σ^2^(*F*_o_^2^) + (0.0098*P*)^2^ + 16.8751*P*] where *P* = (*F*_o_^2^ + 2*F*_c_^2^)/3
6018 reflections	(Δ/σ)_max_ = 0.001
484 parameters	Δρ_max_ = 2.12 e Å^−^^3^
0 restraints	Δρ_min_ = −2.69 e Å^−^^3^

### Fractional atomic coordinates and isotropic or equivalent isotropic displacement parameters (Å^2^)

**Table:**

	*x*	*y*	*z*	*U*_iso_*/*U*_eq_	Occ. (<1)
Pb1	0.72835 (4)	0.74410 (3)	0.64620 (3)	0.04239 (14)	
N1	0.7303 (9)	0.5537 (7)	0.5424 (7)	0.047 (2)	
N2	0.8978 (8)	0.7546 (7)	0.5598 (7)	0.047 (2)	
N3	0.9155 (9)	0.9162 (7)	0.7848 (7)	0.049 (2)	
N4	0.9265 (8)	0.7021 (6)	0.7996 (6)	0.043 (2)	
O1	0.7104 (16)	0.9221 (13)	0.5375 (13)	0.063 (4)	0.60
O2	0.5878 (19)	0.7499 (14)	0.4192 (14)	0.066 (5)	0.60
O1'	0.550 (3)	0.775 (2)	0.429 (2)	0.063 (4)	0.40
O2'	0.644 (3)	0.905 (2)	0.529 (2)	0.066 (5)	0.40
O3	0.6374 (9)	0.7678 (6)	0.7949 (6)	0.064 (2)	
O4	0.6293 (8)	0.5930 (6)	0.7477 (6)	0.057 (2)	
O5	1.3159 (15)	0.8677 (11)	0.4039 (14)	0.063 (5)	0.50
H5A	1.3934	0.8619	0.4441	0.095*	0.50
H5B	1.3075	0.9246	0.4365	0.095*	0.50
O6	0.6197 (9)	0.3730 (6)	0.7780 (6)	0.065 (2)	
H6A	0.5897	0.4227	0.7445	0.097*	
H6B	0.5511	0.3215	0.7351	0.097*	
O7	1.1636 (14)	0.6849 (11)	0.2400 (11)	0.044 (3)	0.50
H7A	1.2270	0.7420	0.2789	0.065*	0.50
H7B	1.1794	0.6245	0.2222	0.065*	0.50
F1	0.507 (2)	1.0371 (15)	0.3788 (13)	0.123 (6)	0.60
F1'	0.751 (3)	0.812 (2)	0.3143 (18)	0.108 (8)	0.40
F2	0.4355 (9)	0.7192 (9)	0.8787 (7)	0.103 (3)	
C1	0.6526 (12)	0.4556 (9)	0.5346 (9)	0.055 (3)	
H1	0.6046	0.4516	0.5753	0.066*	
C2	0.6387 (12)	0.3578 (10)	0.4685 (9)	0.059 (3)	
H2	0.5850	0.2906	0.4675	0.071*	
C3	0.7050 (12)	0.3622 (10)	0.4055 (10)	0.061 (3)	
H3	0.6943	0.2986	0.3589	0.073*	
C4	0.7910 (11)	0.4659 (9)	0.4118 (8)	0.051 (3)	
C5	0.8669 (13)	0.4762 (11)	0.3518 (9)	0.060 (3)	
H5	0.8593	0.4140	0.3052	0.072*	
C6	0.9475 (12)	0.5716 (11)	0.3607 (9)	0.060 (3)	
H6	0.9962	0.5756	0.3205	0.072*	
C7	0.9620 (11)	0.6705 (10)	0.4319 (9)	0.051 (3)	
C8	1.0461 (12)	0.7729 (11)	0.4430 (10)	0.064 (3)	
H8	1.0933	0.7804	0.4016	0.077*	
C9	1.0598 (13)	0.8641 (11)	0.5160 (11)	0.070 (4)	
H9	1.1191	0.9323	0.5272	0.084*	
C10	0.9821 (11)	0.8500 (9)	0.5716 (9)	0.056 (3)	
H10	0.9898	0.9110	0.6197	0.068*	
C11	0.8879 (10)	0.6647 (9)	0.4920 (7)	0.042 (2)	
C12	0.7996 (10)	0.5592 (8)	0.4811 (8)	0.045 (2)	
C13	0.6235 (13)	0.8594 (10)	0.4401 (9)	0.057 (3)	
C14	0.6234 (10)	0.9175 (9)	0.3497 (8)	0.049 (3)	
C15	0.5678 (14)	1.0049 (11)	0.3256 (10)	0.069 (4)	
H15	0.5254	1.0271	0.3658	0.082*	0.40
C16	0.5713 (15)	1.0614 (11)	0.2456 (11)	0.077 (4)	
H16	0.5343	1.1214	0.2327	0.093*	
C17	0.6316 (18)	1.0260 (13)	0.1851 (13)	0.094 (5)	
H17	0.6352	1.0624	0.1300	0.113*	
C18	0.6857 (18)	0.9391 (13)	0.2050 (14)	0.095 (5)	
H18	0.7253	0.9154	0.1631	0.114*	
C19	0.6820 (14)	0.8858 (12)	0.2871 (11)	0.073 (4)	
H19	0.7204	0.8266	0.3004	0.088*	0.60
C21	0.9066 (12)	1.0191 (9)	0.7796 (9)	0.059 (3)	
H21	0.8335	1.0311	0.7226	0.071*	
C22	1.0049 (13)	1.1130 (9)	0.8582 (10)	0.058 (3)	
H22	0.9972	1.1849	0.8520	0.070*	
C23	1.1079 (14)	1.0945 (11)	0.9404 (11)	0.068 (4)	
H23	1.1721	1.1547	0.9926	0.081*	
C24	1.1223 (12)	0.9878 (10)	0.9504 (9)	0.054 (3)	
C25	1.2286 (12)	0.9633 (11)	1.0367 (10)	0.065 (3)	
H25	1.2948	1.0216	1.0901	0.078*	
C26	1.2368 (12)	0.8607 (12)	1.0439 (9)	0.069 (4)	
H26	1.3098	0.8485	1.1008	0.083*	
C27	1.1343 (10)	0.7660 (10)	0.9648 (8)	0.048 (3)	
C28	1.1366 (11)	0.6560 (10)	0.9718 (9)	0.055 (3)	
H28	1.2072	0.6401	1.0281	0.066*	
C29	1.0331 (12)	0.5704 (10)	0.8945 (9)	0.057 (3)	
H29	1.0308	0.4962	0.8982	0.068*	
C30	0.9329 (10)	0.6003 (8)	0.8114 (8)	0.044 (2)	
H30	0.8638	0.5428	0.7592	0.052*	
C31	1.0297 (10)	0.7869 (8)	0.8786 (8)	0.044 (2)	
C32	1.0205 (10)	0.8983 (9)	0.8695 (8)	0.048 (3)	
C33	0.6272 (10)	0.6715 (8)	0.8114 (8)	0.040 (2)	
C34	0.6202 (11)	0.6488 (8)	0.9125 (8)	0.047 (3)	
C35	0.5280 (12)	0.6764 (10)	0.9452 (10)	0.057 (3)	
C36	0.5325 (16)	0.6643 (12)	1.0452 (12)	0.077 (4)	
H36	0.4726	0.6875	1.0663	0.093*	
C37	0.6264 (16)	0.6175 (11)	1.1134 (10)	0.075 (4)	
H37	0.6265	0.6046	1.1789	0.090*	
C38	0.7195 (16)	0.5900 (12)	1.0850 (10)	0.080 (4)	
H38	0.7865	0.5631	1.1333	0.095*	
C39	0.7147 (14)	0.6022 (10)	0.9849 (9)	0.064 (3)	
H39	0.7755	0.5789	0.9650	0.077*	

### Atomic displacement parameters (Å^2^)

**Table:**

	*U*^11^	*U*^22^	*U*^33^	*U*^12^	*U*^13^	*U*^23^
Pb1	0.0453 (2)	0.0421 (2)	0.0347 (2)	0.01148 (17)	0.01311 (17)	0.00643 (15)
N1	0.047 (5)	0.040 (5)	0.047 (5)	−0.001 (4)	0.016 (4)	0.019 (4)
N2	0.042 (5)	0.046 (5)	0.057 (5)	0.004 (4)	0.030 (4)	0.011 (4)
N3	0.061 (6)	0.043 (5)	0.048 (5)	0.018 (4)	0.024 (5)	0.015 (4)
N4	0.053 (5)	0.034 (4)	0.041 (5)	0.010 (4)	0.020 (4)	0.012 (4)
O1	0.076 (10)	0.051 (7)	0.056 (8)	−0.002 (7)	0.031 (8)	0.022 (6)
O2	0.071 (11)	0.057 (8)	0.059 (8)	0.016 (7)	0.019 (7)	0.004 (6)
O1'	0.076 (10)	0.051 (7)	0.056 (8)	−0.002 (7)	0.031 (8)	0.022 (6)
O2'	0.071 (11)	0.057 (8)	0.059 (8)	0.016 (7)	0.019 (7)	0.004 (6)
O3	0.100 (7)	0.042 (4)	0.053 (5)	0.015 (4)	0.038 (5)	0.006 (4)
O4	0.070 (5)	0.050 (4)	0.050 (4)	0.027 (4)	0.021 (4)	0.002 (4)
O5	0.049 (9)	0.031 (7)	0.120 (14)	0.019 (7)	0.041 (9)	0.024 (8)
O6	0.077 (6)	0.050 (5)	0.056 (5)	0.015 (4)	0.021 (4)	0.008 (4)
O7	0.058 (9)	0.045 (8)	0.059 (8)	0.035 (7)	0.043 (7)	0.022 (7)
F1	0.173 (17)	0.140 (15)	0.122 (13)	0.114 (14)	0.085 (13)	0.065 (11)
F1'	0.14 (2)	0.14 (2)	0.115 (17)	0.108 (18)	0.086 (16)	0.063 (15)
F2	0.092 (6)	0.136 (8)	0.105 (7)	0.056 (6)	0.052 (5)	0.041 (6)
C1	0.061 (7)	0.057 (7)	0.046 (6)	0.013 (6)	0.022 (6)	0.018 (5)
C2	0.057 (7)	0.044 (6)	0.059 (7)	0.008 (6)	0.011 (6)	0.016 (6)
C3	0.066 (8)	0.042 (6)	0.056 (7)	0.013 (6)	0.011 (6)	0.004 (5)
C4	0.046 (6)	0.053 (7)	0.030 (5)	0.010 (5)	−0.001 (5)	0.001 (5)
C5	0.067 (8)	0.062 (8)	0.051 (7)	0.022 (7)	0.026 (6)	0.002 (6)
C6	0.058 (7)	0.084 (9)	0.042 (6)	0.019 (7)	0.026 (6)	0.006 (6)
C7	0.047 (6)	0.059 (7)	0.049 (6)	0.015 (6)	0.022 (5)	0.011 (5)
C8	0.052 (7)	0.083 (9)	0.056 (7)	0.004 (7)	0.028 (6)	0.012 (7)
C9	0.073 (9)	0.058 (8)	0.079 (9)	−0.001 (7)	0.041 (8)	0.017 (7)
C10	0.056 (7)	0.044 (6)	0.059 (7)	−0.003 (5)	0.026 (6)	−0.001 (5)
C11	0.043 (6)	0.050 (6)	0.032 (5)	0.018 (5)	0.012 (4)	0.006 (4)
C12	0.049 (6)	0.042 (6)	0.040 (6)	0.008 (5)	0.017 (5)	0.016 (5)
C13	0.068 (8)	0.053 (7)	0.039 (6)	0.020 (6)	0.011 (6)	0.012 (5)
C14	0.039 (6)	0.043 (6)	0.040 (6)	0.005 (5)	−0.002 (5)	0.001 (5)
C15	0.069 (8)	0.065 (8)	0.061 (8)	0.028 (7)	0.016 (7)	0.001 (7)
C16	0.077 (9)	0.052 (8)	0.069 (9)	0.013 (7)	−0.001 (8)	0.023 (7)
C17	0.134 (15)	0.070 (10)	0.089 (11)	0.019 (10)	0.057 (11)	0.049 (9)
C18	0.126 (14)	0.082 (11)	0.110 (13)	0.018 (10)	0.085 (12)	0.034 (10)
C19	0.082 (10)	0.073 (9)	0.081 (9)	0.026 (8)	0.046 (8)	0.025 (7)
C21	0.062 (7)	0.053 (7)	0.054 (7)	0.020 (6)	0.015 (6)	0.012 (6)
C22	0.074 (8)	0.028 (5)	0.064 (8)	−0.004 (5)	0.031 (7)	0.006 (5)
C23	0.071 (9)	0.057 (8)	0.065 (8)	0.009 (7)	0.028 (7)	−0.009 (6)
C24	0.057 (7)	0.052 (7)	0.057 (7)	0.009 (6)	0.032 (6)	0.004 (5)
C25	0.057 (8)	0.058 (8)	0.050 (7)	−0.002 (6)	0.007 (6)	−0.004 (6)
C26	0.045 (7)	0.100 (11)	0.033 (6)	0.017 (7)	−0.007 (5)	−0.004 (6)
C27	0.040 (6)	0.064 (7)	0.034 (5)	0.019 (5)	0.008 (5)	0.004 (5)
C28	0.048 (6)	0.070 (8)	0.041 (6)	0.026 (6)	0.008 (5)	0.012 (6)
C29	0.063 (7)	0.059 (7)	0.054 (7)	0.028 (6)	0.023 (6)	0.021 (6)
C30	0.040 (5)	0.044 (6)	0.033 (5)	0.004 (5)	0.008 (4)	−0.001 (4)
C31	0.048 (6)	0.043 (6)	0.033 (5)	0.006 (5)	0.016 (5)	−0.005 (4)
C32	0.042 (6)	0.043 (6)	0.045 (6)	0.003 (5)	0.015 (5)	−0.013 (5)
C33	0.047 (6)	0.039 (6)	0.039 (5)	0.009 (5)	0.024 (5)	0.009 (4)
C34	0.056 (6)	0.029 (5)	0.047 (6)	0.001 (5)	0.021 (5)	0.002 (4)
C35	0.055 (7)	0.057 (7)	0.073 (8)	0.015 (6)	0.039 (6)	0.030 (6)
C36	0.102 (11)	0.078 (9)	0.093 (10)	0.035 (9)	0.075 (9)	0.030 (8)
C37	0.112 (12)	0.061 (8)	0.047 (7)	0.009 (8)	0.038 (8)	0.001 (6)
C38	0.102 (11)	0.087 (10)	0.054 (8)	0.038 (9)	0.033 (8)	0.018 (7)
C39	0.101 (10)	0.058 (7)	0.052 (7)	0.024 (7)	0.045 (7)	0.029 (6)

### Geometric parameters (Å, °)

**Table:**

Pb1—N1	2.675 (9)	C8—H8	0.9300
Pb1—N2	2.644 (8)	C9—C10	1.387 (17)
Pb1—N3	2.622 (9)	C9—H9	0.9300
Pb1—N4	2.566 (8)	C10—H10	0.9300
Pb1—O1	2.788 (16)	C11—C12	1.442 (15)
Pb1—O1'	2.95 (3)	C13—C14	1.496 (16)
Pb1—O2	2.880 (18)	C14—C19	1.368 (17)
Pb1—O2'	2.77 (3)	C14—C15	1.372 (15)
Pb1—O3	2.670 (8)	C15—C16	1.37 (2)
Pb1—O4	2.777 (9)	C15—H15	0.9300
N1—C1	1.326 (14)	C16—C17	1.38 (2)
N1—C12	1.372 (14)	C16—H16	0.9300
N2—C10	1.321 (14)	C17—C18	1.35 (2)
N2—C11	1.351 (12)	C17—H17	0.9300
N3—C21	1.317 (13)	C18—C19	1.37 (2)
N3—C32	1.359 (13)	C18—H18	0.9300
N4—C30	1.310 (12)	C19—H19	0.9300
N4—C31	1.387 (12)	C21—C22	1.430 (15)
O1—C13	1.340 (19)	C21—H21	0.9300
O2—C13	1.32 (2)	C22—C23	1.332 (17)
O1'—C13	1.16 (3)	C22—H22	0.9300
O2'—C13	1.22 (3)	C23—C24	1.389 (17)
O3—C33	1.237 (12)	C23—H23	0.9300
O4—C33	1.269 (11)	C24—C25	1.415 (16)
O5—H5A	0.85	C24—C32	1.423 (15)
O5—H5B	0.85	C25—C26	1.314 (17)
O6—H6A	0.85	C25—H25	0.9300
O6—H6B	0.85	C26—C27	1.454 (16)
O7—H7A	0.85	C26—H26	0.9300
O7—H7B	0.85	C27—C31	1.383 (14)
F1—C15	1.289 (19)	C27—C28	1.393 (15)
F1'—C19	1.32 (2)	C28—C29	1.385 (16)
F2—C35	1.326 (13)	C28—H28	0.9300
C1—C2	1.398 (15)	C29—C30	1.388 (14)
C1—H1	0.9300	C29—H29	0.9300
C2—C3	1.364 (18)	C30—H30	0.9300
C2—H2	0.9300	C31—C32	1.432 (14)
C3—C4	1.429 (17)	C33—C34	1.475 (14)
C3—H3	0.9300	C34—C35	1.394 (15)
C4—C12	1.401 (14)	C34—C39	1.402 (16)
C4—C5	1.419 (17)	C35—C36	1.379 (17)
C5—C6	1.311 (18)	C36—C37	1.376 (19)
C5—H5	0.9300	C36—H36	0.9300
C6—C7	1.445 (15)	C37—C38	1.37 (2)
C6—H6	0.9300	C37—H37	0.9300
C7—C8	1.389 (17)	C38—C39	1.380 (17)
C7—C11	1.401 (15)	C38—H38	0.9300
C8—C9	1.389 (17)	C39—H39	0.9300
			
N4—Pb1—N3	63.5 (3)	C9—C10—H10	118.2
N4—Pb1—N2	79.2 (3)	N2—C11—C7	121.9 (10)
N3—Pb1—N2	82.0 (3)	N2—C11—C12	119.4 (9)
N4—Pb1—O3	84.9 (3)	C7—C11—C12	118.7 (9)
N3—Pb1—O3	79.9 (3)	N1—C12—C4	122.4 (10)
N2—Pb1—O3	159.9 (3)	N1—C12—C11	118.3 (9)
N4—Pb1—N1	81.0 (2)	C4—C12—C11	119.3 (10)
N3—Pb1—N1	133.9 (3)	O1'—C13—O2'	101 (2)
N2—Pb1—N1	62.4 (3)	O2'—C13—O2	116.8 (18)
O3—Pb1—N1	127.2 (3)	O1'—C13—O1	123.3 (17)
N4—Pb1—O2'	144.5 (6)	O2—C13—O1	124.4 (14)
N3—Pb1—O2'	82.9 (6)	O1'—C13—C14	124.9 (17)
N2—Pb1—O2'	85.4 (6)	O2'—C13—C14	125.0 (16)
O3—Pb1—O2'	100.9 (6)	O2—C13—C14	117.9 (12)
N1—Pb1—O2'	119.4 (6)	O1—C13—C14	111.5 (12)
N4—Pb1—O4	71.7 (3)	C19—C14—C15	116.4 (12)
N3—Pb1—O4	112.5 (2)	C19—C14—C13	120.6 (10)
N2—Pb1—O4	135.2 (2)	C15—C14—C13	123.0 (12)
O3—Pb1—O4	47.5 (2)	F1—C15—C16	116.5 (14)
N1—Pb1—O4	79.8 (2)	F1—C15—C14	119.9 (15)
O2'—Pb1—O4	136.6 (6)	C16—C15—C14	123.6 (14)
N4—Pb1—O1	131.7 (4)	C16—C15—H15	118.2
N3—Pb1—O1	74.0 (4)	C14—C15—H15	118.2
N2—Pb1—O1	73.0 (4)	C15—C16—C17	117.6 (13)
O3—Pb1—O1	110.0 (4)	C15—C16—H16	121.2
N1—Pb1—O1	117.3 (4)	C17—C16—H16	121.2
O2'—Pb1—O1	14.6 (6)	C18—C17—C16	120.7 (14)
O4—Pb1—O1	150.7 (4)	C18—C17—H17	119.7
N4—Pb1—O2	148.7 (4)	C16—C17—H17	119.7
N3—Pb1—O2	121.6 (4)	C17—C18—C19	120.0 (15)
N2—Pb1—O2	71.7 (4)	C17—C18—H18	120.0
O3—Pb1—O2	126.0 (4)	C19—C18—H18	120.0
N1—Pb1—O2	75.6 (3)	F1'—C19—C14	119.0 (15)
O2'—Pb1—O2	44.9 (6)	F1'—C19—C18	118.6 (16)
O4—Pb1—O2	123.1 (4)	C14—C19—C18	121.8 (13)
O1—Pb1—O2	48.9 (5)	C14—C19—H19	119.1
N4—Pb1—O1'	160.8 (6)	C18—C19—H19	119.1
N3—Pb1—O1'	119.0 (6)	N3—C21—C22	122.6 (10)
N2—Pb1—O1'	82.3 (6)	N3—C21—H21	118.7
O3—Pb1—O1'	114.3 (6)	C22—C21—H21	118.7
N1—Pb1—O1'	85.9 (6)	C23—C22—C21	118.1 (11)
O2'—Pb1—O1'	37.3 (7)	C23—C22—H22	121.0
O4—Pb1—O1'	119.8 (6)	C21—C22—H22	121.0
O1—Pb1—O1'	45.0 (6)	C22—C23—C24	121.9 (11)
O2—Pb1—O1'	12.3 (6)	C22—C23—H23	119.1
C1—N1—C12	117.9 (9)	C24—C23—H23	119.1
C1—N1—Pb1	123.0 (8)	C23—C24—C25	124.2 (11)
C12—N1—Pb1	118.4 (6)	C23—C24—C32	117.0 (11)
C10—N2—C11	118.8 (9)	C25—C24—C32	118.8 (11)
C10—N2—Pb1	120.9 (7)	C26—C25—C24	122.1 (11)
C11—N2—Pb1	119.8 (7)	C26—C25—H25	118.9
C21—N3—C32	118.8 (9)	C24—C25—H25	118.9
C21—N3—Pb1	122.2 (7)	C25—C26—C27	121.4 (10)
C32—N3—Pb1	118.8 (6)	C25—C26—H26	119.3
C30—N4—C31	116.2 (8)	C27—C26—H26	119.3
C30—N4—Pb1	122.4 (6)	C31—C27—C28	118.8 (10)
C31—N4—Pb1	121.3 (6)	C31—C27—C26	118.0 (10)
C13—O1—Pb1	94.8 (9)	C28—C27—C26	123.2 (10)
C13—O2—Pb1	91.3 (10)	C29—C28—C27	119.6 (9)
C13—O1'—Pb1	91.0 (17)	C29—C28—H28	120.2
C13—O2'—Pb1	98.8 (15)	C27—C28—H28	120.2
C33—O3—Pb1	96.9 (6)	C28—C29—C30	117.1 (10)
C33—O4—Pb1	91.0 (6)	C28—C29—H29	121.5
H5A—O5—H5B	105.4	C30—C29—H29	121.5
H6A—O6—H6B	92.6	N4—C30—C29	126.1 (10)
H7A—O7—H7B	121.0	N4—C30—H30	117.0
N1—C1—C2	123.7 (12)	C29—C30—H30	117.0
N1—C1—H1	118.1	C27—C31—N4	122.3 (10)
C2—C1—H1	118.1	C27—C31—C32	121.0 (9)
C3—C2—C1	119.2 (12)	N4—C31—C32	116.7 (9)
C3—C2—H2	120.4	N3—C32—C24	121.6 (10)
C1—C2—H2	120.4	N3—C32—C31	119.7 (9)
C2—C3—C4	119.2 (11)	C24—C32—C31	118.7 (10)
C2—C3—H3	120.4	O3—C33—O4	122.5 (9)
C4—C3—H3	120.4	O3—C33—C34	118.6 (9)
C12—C4—C5	119.9 (11)	O4—C33—C34	118.8 (9)
C12—C4—C3	117.6 (11)	C35—C34—C39	116.1 (11)
C5—C4—C3	122.5 (11)	C35—C34—C33	123.8 (10)
C6—C5—C4	121.5 (11)	C39—C34—C33	119.9 (10)
C6—C5—H5	119.3	F2—C35—C36	119.3 (11)
C4—C5—H5	119.3	F2—C35—C34	118.3 (11)
C5—C6—C7	121.1 (11)	C36—C35—C34	122.4 (12)
C5—C6—H6	119.4	C37—C36—C35	119.4 (12)
C7—C6—H6	119.4	C37—C36—H36	120.3
C8—C7—C11	117.9 (10)	C35—C36—H36	120.3
C8—C7—C6	122.6 (11)	C38—C37—C36	120.1 (13)
C11—C7—C6	119.5 (11)	C38—C37—H37	120.0
C9—C8—C7	119.9 (12)	C36—C37—H37	120.0
C9—C8—H8	120.0	C37—C38—C39	120.3 (13)
C7—C8—H8	120.0	C37—C38—H38	119.8
C10—C9—C8	117.7 (12)	C39—C38—H38	119.8
C10—C9—H9	121.1	C38—C39—C34	121.5 (13)
C8—C9—H9	121.1	C38—C39—H39	119.3
N2—C10—C9	123.6 (11)	C34—C39—H39	119.3
N2—C10—H10	118.2		

### Hydrogen-bond geometry (Å, °)

**Table:**

*D*—H···*A*	*D*—H	H···*A*	*D*···*A*	*D*—H···*A*
O5—H5A···O1'^i^	0.85	2.34	3.027 (4)	138
O5—H5A···F1^ii^	0.85	2.33	2.801 (5)	116
O5—H5A···O2'^i^	0.85	2.51	3.313 (6)	158
O5—H5B···O2'^ii^	0.85	2.05	2.789 (3)	146
O5—H5B···O1^ii^	0.85	1.99	2.792 (6)	158
O6—H6A···O4	0.85	2.08	2.807 (11)	143
O6—H6B···O2^iii^	0.85	2.03	2.795 (5)	149
O6—H6B···O1'^iii^	0.85	2.17	2.889 (5)	143
O7—H7A···O5	0.85	1.97	2.75 (2)	152
O7—H7B···O6^iv^	0.85	2.29	2.810 (2)	120
C8—H8···O5	0.93	2.54	3.344 (34)	145
C16—H16···O3^v^	0.93	2.54	3.422 (19)	158
C21—H21···O1	0.93	2.44	3.106 (82)	127

Symmetry codes: (i) *x*+1, *y*, *z*; (ii) −*x*+2, −*y*+2, −*z*+1;  (iii) −*x*+1, −*y*+1, −*z*+1; (iv) −*x*+2, −*y*+1, −*z*+1; (v) −*x*+1, −*y*+2, −*z*+1.
